# Grain Diversity Effects on Banker Plant Growth and Parasitism by *Aphidius colemani*

**DOI:** 10.3390/insects6030772

**Published:** 2015-09-08

**Authors:** Travis McClure, Steven D. Frank

**Affiliations:** Department of Entomology, North Carolina State University, Campus Box 7613, Raleigh, NC 27695, USA; E-Mail: tjmcclur@ncsu.edu

**Keywords:** banker plant, monoculture *vs.* mixture, *Aphidius colemani*, aphid biological control

## Abstract

Green peach aphid (*Myzus persicae* Sulzer) (Hemiptera: Aphididae) is a serious greenhouse pest with a short generation time, parthenogenetic reproduction and a broad host range. Banker plant systems are becoming a more common form of biological control for this pest. This system consists of grain “banker plants” infested with *R. padi*, an alternative hosts for the parasitoid *Aphidius colemani*. Thus *A. colemani* can reproduce on the banker plant when *M. persicae* populations are low. This system can increase pest suppression; however, like other biological control tools, efficacy is inconsistent. One reason is because several different grain species have been used. Our studies determined if there were benefits to planting interspecific mixture banker plants, similar to when open agricultural systems use mixed cropping. Our study found that although banker plants grow larger when planted as mixtures this added plant growth does not increase in the number of aphids, or mummies an individual banker plant can sustain. Rye banker plants grew larger, and sustained more mummies than the other species we tested, but barley banker plants resulted in a similar number of aphids in a more condensed area. Ultimately, we did not see any differences in pest suppression between monoculture banker plants, mixture banker plants, or our augmentative release treatment. However, using banker plants resulted in more female parasitoids than the augmentative release, a benefit to using banker plant systems.

## 1. Introduction

Increasing plant diversity can be an effective conservation biological control strategy in many cropping systems. Plant diversity can increase insect natural enemy abundance, fitness, and efficacy by providing alternative hosts and food resources [1,2,3]. Natural enemies are scarcer in greenhouses than outdoor cropping systems because greenhouse construction excludes them. In addition, greenhouses often contain a monoculture or low crop diversity which may not provide sufficient resources for natural enemies. Banker plant systems are a conservation biological control strategy for greenhouses which increase plant diversity and provide resources for natural enemies [4,5]. The goal of a banker plant system is to support a reproducing natural enemy population within a crop to provide pest suppression for an extended period [4].

Banker plants are typically non-crop plants grown within a greenhouse that provide natural enemies with alternative food, such as pollen, or alternative hosts for parasitoids [4,6,7]. The most common banker plant system consists of grain “banker” plants infested with *Rhopalosiphum padi* L. (Hemiptera: Aphididae). Since *R. padi* only feed on monocots, they are not pests of most greenhouse crops. *Rhopalosiphum padi* are hosts for the parasitoid *Aphidius colemani* Viereck (Hymenoptera: Braconidae) commonly used to suppress important greenhouse pests *Aphis gossypii* Glover and *Myzus persicae* Sulzer (Hemiptera: Aphididae). By reproducing on *R. padi*, *A. colemani* can maintain a population within a greenhouse even when pest abundance is low. Studies on this system have shown that banker plants can increase parasitism and reduce pest aphid populations compared to greenhouses without banker plants [8].

Plants can affect parasitoid fitness by influencing the quality of their insect hosts [9]. Plant species or varieties that are poor quality for herbivores due to allelochemicals or poor nutrition can reduce parasitoid fitness [10,11,12]. For example, aphid resistant soybean varieties have been shown to reduce parasitoid development [13]. At least eight different grain species have been used as banker plants to maintain *R. padi* [4]. The effects of all these species on parasitoid fitness is not known but a recent study found that *A. colemani* populations reared on aphids fed on barley, oats, rye, or wheat each expressed a different suite of life history characteristics such as sex ratio [14]. Theory predicts diverse natural enemy communities or phenotypes should increase pest suppression [15,16]. Since *A. colemani* life history characters vary among the grain species on which their natal hosts fed, an interspecific mixture that produces a variety of phenotypes may increase pest suppression by *A. colemani* in greenhouses.

In aphid banker plant systems, using an interspecific grain mixture could have several advantages or disadvantages compared to monoculture banker plants. For example, greater plant growth could increase *R. padi* population size and support more *A. colemani* [17,18,19]. This could be a disadvantage if mixtures grow too large or dense and become a nuisance to growers or make it difficult for parasitoids to find *R. padi* [20,21]. Plant species mixtures could reduce aphid population growth due to associational resistance [3,22]. This could be advantageous if reducing *R. padi* abundance on banker plants increased the banker plant lifespan, and thus reduced replacement frequency. In addition, *A. colemani* are attracted to denser aphid populations [17,23]. Reducing the number of aphids present on banker plants may encourage *A. colemani* to oviposit on the potentially denser pest aphid populations. On the other hand, if grain mixtures reduce aphid abundance on banker plants, this could reduce the number of hosts available for *A. colemani* and thus reduce parasitoid abundance. Plant diversity can also change plant volatile and visual cues which increase attractiveness to parasitoids [24,25]. In our aphid banker plant system this could be an advantage if it helps retain *A. colemani* within the greenhouse when pest abundance is low. However, increased banker plant attractiveness could be a disadvantage if *A. colemani* preferentially oviposit on the banker plants rather than on the crop, reducing pest suppression. Alternatively, since *A. colemani* reared on different grain species have different attributes, such as size, they could increase pest suppression by targeting different aphid instars or sizes [26]. However, research on the closely related *A. ervi* suggests larger parasitoids are able to overcome aphid defensive behaviors and parasitize aphids of all instars and sizes [27]. Further, Lin and Ives [28], suggest *A. colemani* prefer larger aphids which also reproduce the most. Diverse *A. colemani* phenotypes could be a disadvantage because not every individual is able target all aphid life stages, particularly the largest and most economically important.

Our objective was to determine if interspecific mixture banker plants increase parasitoid fitness and pest suppression compared to monoculture banker plants in an aphid banker plant system. We used greenhouse experiments to determine how grain mixtures affect: (1) banker plant growth characteristics; (2) the duration and number of *R. padi* and *A. colemani* the system produced; (3) *A. colemani* preference for *R. padi* on banker plants compared to *M. persicae* on the crop; and (4) *A. colemani* pest suppression and fitness. These results will help determine if growers should be advised to use mixture banker plants over traditional monoculture banker plants.

## 2. Materials and Methods

### 2.1. Study System

For all experiments, we used Black Pearl pepper plants (*Capsicum anuum* L. “Black Pearl”) infested with the pest aphid *M. persicae* as crop plants. We propagated pepper plants from cuttings. We cut source plants roughly 5 cm below the bud. Then we dipped cut tips into “Rhizopon AA Dry Powder Rooting Hormone #1” (Active ingredient: 0.1% 3-Indolebuteric acid) (Earth City, MO, USA) and potted them into a sifted Fafard 2P mix (Agawam, MA). We planted cuttings in 48 cell trays (56 cm by 25.5 cm tray), and allowed them to root for 6 weeks before we transplanted each into separate 15.2 cm-diameter pots containing Fafard 2P soil mix and 8.86 g of Scotts Osmocote (N-P-K: 14-14-14) fertilizer (Marysville, OH, USA) per pot. We only used plants between 25 cm and 40 cm tall.

We maintained aphid colonies in the laboratory from field-collected aphids. Our *M. persicae* were reared on pepper plants. Our *R. padi* colonies were reared in cages containing monoculture and mixture pots of the same grain species and cultivars used in experiments (Table 1). We originally purchased *Aphidius colemani*, from Koppert Biological (Howell, MI); and then maintained them in colony in cages with *R. padi* on the same monoculture and mixture grain plants as we used in experiments. We kept all colonies in separate 60 × 60 × 60 cm BugDorm cages (BugDorm Store; Taipei, Taiwan) for two years in a greenhouse receiving ambient light with temperatures ranging from 18.5 °C to 34.5 °C depending on season.

**Table 1:** Species and cultivars composition for the monoculture and mixture banker plant treatments.

Monoculture		Mixture			
		Barley	Oat	Rye	Wheat
**Barley**	Price	Price	Cat grass	Wrens Abrozzi	Roane
	Thoroughbred	Nomini	Cat grass	Matt time	USG 3209
	Nomini	Nomini	Cat grass	Wrens Abrozzi	Neuse
**Oat**	Cat grass	Thoroughbred	Rodgers	Matt time	USG 3209
	Brooks	Price	Brooks	Wrens Abrozzi	Neuse
	Rodgers	Price	Rodgers	AGS 104	Roane
**Rye**	Matt time	Nomini	Brooks	AGS 104	USG 3209
	Wrens Abrozzi	Thoroughbred	Cat grass	Wrens Abrozzi	Neuse
	AGS 104	Thoroughbred	Rodgers	Matt time	Roane
**Wheat**	Roane	Thoroughbred	Brooks	AGS 104	Neuse
	USG 3209	Nomini	Cat grass	Wrens Abrozzi	Roane
	Neuse	Thoroughbred	Brooks	AGS 104	USG 3209

### 2.2. Banker Plant Growth Characteristics

To understand how plant species and mixtures affect banker plant growth we conducted a greenhouse experiment with the four most common aphid banker plant species barley, oat, rye, and wheat [4]. Barley, oat, rye, and wheat were all tested as monocultures. Within each species we used 3 different cultivars so the effect of species was not determined by a single cultivar. The species and cultivars used were barley, *Hordeum vulgare* L. (“Price”, “Thoroughbred”, and “Nomni”), oat, *Avena sativa* L. (“Cat Grass”, “Brooks”, and “Rodgers”), rye, *Secale cereale* L. (“Matt time”, “Wrens Abrozzi”, and “AGS 104”), and winter wheat, *Triticum aestivum* L. (“Roane”, “USG 3209/Jaypee”, and “Neuse”) (Table 1). We planted three replicate pots of each cultivar of each plant species. This provided 36 pots of the monoculture treatment to compare the overall effects of growing banker plants in monocultures to growing them as mixtures. It also provided 9 pots of each species within the monoculture treatment to determine if the species differed in their growth characteristics. The mixture treatment was made up of 12 unique four species mixtures, containing one randomly selected cultivar from each of the four species, used in the monoculture treatment (*n* = 3 each), planted in equal proportions (6 seeds each) for a total of 36 mixture pots. We planted all treatments with 24 seeds unsystematically arranged in 15.2 cm-diameter pots with potting mix (2 mix; Fafard^®^, Agawam, MA, USA) and 8.86 g of Osmocote^®^ (N-P-K: 14-14-14) fertilizer (The Scotts Company LLC, Marysville, OH, USA).

We unsystematically placed the pots onto two 2.4 × 1.2 m greenhouse bench sections arranged in 5 rows with 14 or 15 pots per row. Pots were staggered with 15 cm diagonally between each. Starting twelve days after planting, we measured four plant growth characters for each pot twice a week for twelve weeks. We counted the number of live plants in each pot. If a pot had no live plants we did not include the pot in the other measures. We measured the height from the top of the soil to the tip of the tallest plant in a given pot. We measured the straightened length of three random leaves per pot from leaf-tip to the node. We calculated the area of plant material in each pot by measuring the widest expanse of plant material from a pot as viewed from above and the width perpendicular to this measurement, adding them together and dividing by four to get the average radius. We then squared this number and multiplied the product by π. We added 30 *R. padi* of mixed instars (randomly drawn from a colony that contained all instars) to each pot eighteen days after planting. Although parasitoids were not released in this experiment, our pots were uncaged and exposed to resident parasitoids.

To determine how banker plant treatment (monoculture or mixture) or species (barley, oat, rye, and wheat) and day affected each of the four plant growth characters, we used 2-way repeated measures ANOVA. Banker plant treatment and day were fixed independent variables while the four plant growth characters were dependent variables, date was the repeated measure with the subject of replicate nested within treatment (*n* = 36), which was also included as a random factor. For species level analyses, banker plant species were fixed independent variables while the four plant growth characters were dependent variables, date was the repeated measure with the subject of replicate nested within treatment (*n* = 9) which was also included as a random factor. The mixture treatment was also included for comparisons (*n* = 36). These tests were also carried out at the cultivar level but there were no significant differences. In each analysis, data for all characters other than number of live plants per plot were log(x + 1) transformed to meet the assumptions of normality. We performed these statistical analyses in SAS (version 9.2, SAS Institute, Inc., Cary, NC, USA, 2009), using the Mixed procedure.

### 2.3. Effect of Interspecific Grain Mixtures on Aphid and Parasitoid Population Growth

Within the same experiment we also observed how *R. padi* and *A. colemani* responded to monoculture and mixture grain plants. To determine the effective duration of a banker plant, we recorded the percentage of banker plants per treatment with more than 10 aphids, the minimum number of aphids at which a banker plant still has the potential to provide parasitoids to the banker plant system. The ten aphids was used as a minimum effective density, because although *A. colemani* perform well at low aphid densities, some aphidaphagous species will not feed on a plant if aphid density is less than 10 [29,30].

We counted aphids and parasitoid mummies in each pot twice per week for four weeks. Aphid and mummy counts were cumulative and represent the total number of aphids and mummies on a given banker plant. These numbers will be referred to as the number of aphids and mummies “produced” by an individual banker plant. Mummy counts were continued for one more session (3 days later) to include the most recent aphids converted to mummies.

We used two-way repeated measures ANOVA to determine how the independent variables, banker plant treatment (monoculture and mixture), days after introduction of *R. padi*, and their interaction affected dependent variables of banker plant effective duration, cumulative *R. padi* and mummy abundance. Days after introduction of *R. padi* was the repeated measure. The subject was pot, nested within treatment (*n* = 36), which was also included as a random variable. We also compared the mixture treatment to the individual monoculture species. We used two-way repeated measures ANOVA to determine how the independent variables banker plant species (barley, oat, rye, wheat, and mixture), days after introduction of *R. padi* and their interaction affected the dependent variables cumulative *R. padi* and mummy abundance. Days after introduction of *R. padi* were the repeated measure. The subject was pot nested within species (*n* = 9, for the monocultures and *n* = 36 for mixture), which was also included as a random variable. We further analyzed significant ANOVA results with pair wise comparisons using Tukey’s HSD. Data were not normally distributed so we log(x + 1) transformed the number of aphids and mummies before analysis. We performed these statistical analyses in SAS (version 9.2) using the proc mixed procedure.

### 2.4. Mixture Banker Plant Effects on A. colemani Host Preference

To determine if mixture banker plants change *A. colemani* host preference between *R. padi* and *M. persicae* we performed a two × two factorial choice assay. We crossed two *A. colemani* rearing habitats, *M. persicae* on pepper and *R. padi* on grain, with two banker plant treatments, monoculture or mixture. We conducted this experiment in 48, 61 × 61 × 61 cm cages, built using PVC pipe and organza, each of which contained one crop plant and one banker plant. Each cage was assigned to one of the four treatment combinations, resulting in 12 possible replicates of treatment combination. We infested crop plants and banker plants with 30 *M. persicae* and *R. padi* respectively. Two days later we released one female parasitoid into each cage. Three hours after releasing the parasitoids we collected them from their cages using an aspirator. In cages where we could not find the parasitoid we hung a yellow sticky trap for 24 h to collect the parasitoid. If no parasitoid was recovered from a cage after 24 h, it was not included in the analysis. One week after the release and collection of the parasitoids we inspected the crop and banker plants and recorded the number of mummies on each. If no parasitism occurred, cages were not included in the analysis. This experiment was repeated twice, resulting in 24 possible replicates for each of the four treatment combinations, while observed replicates ranged from 13 to 18 (14 grain reared monoculture; 18 grained reared mixture; 15 pepper reared monoculture; 13 pepper reared mixture).

We used 2-way ANOVA to determine how the independent variables natal habitat (*M. persicae* on pepper and *R. padi* on grain), banker plant treatment (monoculture and mixture), and the interaction affected the dependent variable of where and how often *A. colemani* oviposited. Time block, location in the greenhouse, and collection method (aspirator 3 h or sticky card 24 h) were included as random variables. Data for both aphids and mummies were not normal, so we log(x + 1) transformed them before analysis. We performed these statistical analyses in SAS (version 9.2) the proc mixed procedure.

### 2.5. Effects of Banker Plant Mixtures on Aphidius colemani Pest Suppression and Fitness

To compare the overall efficacy of monoculture and mixture banker plants in a cage experiment we used two banker plant treatments (monoculture and mixture) as in Section 2.3 and one “industry control” treatment which consisted of one augmentative release of *A. colemani* but no banker plant. We used 61 × 61 × 61 cm organza cages each containing one pepper crop plant and one of the banker plant treatments. For this experiment we had 12 monoculture (3 cultivars of each of the four species) and 12 mixture cages (each of the 12 mixture combinations), and 12 augmentative cages for 36 cages total. On 5 October 2012 we planted banker plants, which became infested with *R. padi* of mixed sizes and instars. On 8 November 2012, we trimmed each grain to a height of 10 cm and aphids were removed until approximately 30 remained. Also on 8 November 2012 (one day prior to the start of the experiment) we placed 30 *M. persicae* on the crop plants. On 9 November 2012 parasitoids were released into the cages via petri dishes placed in the center of each cage. Parasitoids were purchased from Koppert Biological. The first four cages of each treatment received ten adult female parasitoids. The remaining eight cages of each treatment received 20 mummies, since we did not have enough adults. This was based on the assumption of a 50:50 sex ratio so each cage that received mummies would have 10 adult females. We added parasitoids to cages sequentially; monoculture 1, mixture 1, augmentative 1, monoculture 2, mixture 2, augmentative 2, *etc.*

Ten, twenty, and twenty-seven days after releasing parasitoids, we counted the number of aphids and mummies on each plant (19 November 2012, 29 November 2012, and 6 December 2012). The final count was made seven days after the previous count instead of ten days because the health of several banker plants was declining. We used the number of *M. persicae* on the crop plant as a measure of pest suppression. *Rhopalosiphum padi*, mummies on banker plants, and mummies on the crop were also recorded in order to determine how monoculture or mixture banker plants affected the density dependent response by *A. colemani*. We could not readily distinguish between eclosed and non-eclosed mummies or remove eclosed mummies without disturbing the plants and aphids. Therefore we counted the total number of mummies on each sampling date and estimated new mummies by subtracting mummies from the previous count.

After completion of the last count, we collected mummies (when present) from each banker plant and each crop plant separately. Mummies were kept in petri dishes for 10 days until adults eclosed. We determined the sex of each parasitoid and measured hind tibia length (mm) as a proxy of adult parasitoid size [31].

We used a two-way ANOVA to determine how the independent variables of banker plant treatment (monoculture and mixture), days after parasitoid release, and their interaction affected the dependent variable of the number of *M. persicae*. These data were not normal so we square root transformed them for analysis. Whether cages received adults or mummies on the initial release was included as a random factor, after there was not a significant effect when the model included it as a fixed effect. To determine if parasitism of *M. persicae* was related to the percentage *M. persicae* in a cage we tested the correlation of the percentage of mummies on the crop within a cage with percentage of aphids that were *M. persicae* using separate Spearman’s correlation for monoculture and mixture banker plant treatments on each of the three data collection days. Control cages were not included in correlation analyses because 100% of the in cage aphids and mummies were *M. persicae*.

For each banker plant treatment we combined the frequency of female wasps from both plants in each cage and used a Chi squared test to calculate the differences between the cage totals. We analyzed size for emerging parasitoids collected off of banker plants and crop plants, but since the controls did not have banker plants we could not compare the size of females collected on banker plants to the size of females on crop plants. We used separate ANOVA to analyze the effect of banker plant treatment on size of parasitoids emerging banker plants and crop plants. We performed these statistical analyses in SAS (version 9.2) using Mixed procedure, Corr procedure, the Freq procedure, and again the Mixed procedures respectively.

## 3. Results

### 3.1. Banker Plant Growth Characteristics

**Figure 1:**
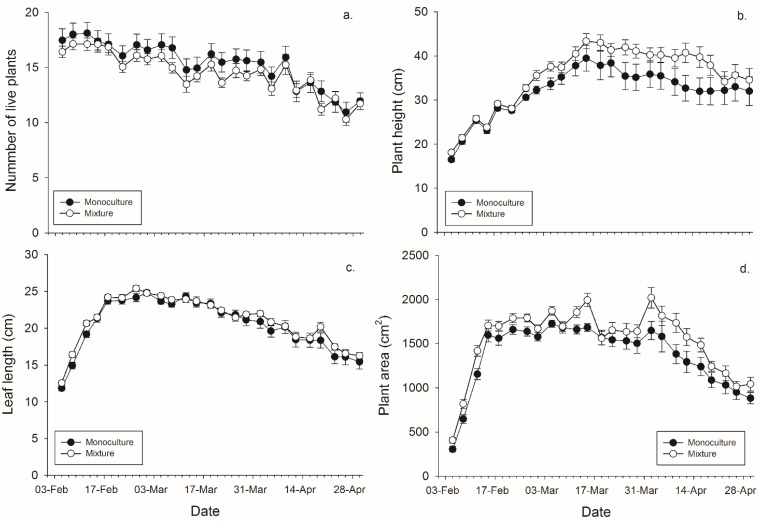
Mean (± SEM) (**a**) number of plants surviving within a pot; (**b**) height of the tallest plant per pot; (**c**) length of the leaves per pot, and (**d**) plant area in monoculture and mixture grain pots for each collection day.

**Figure 2:**
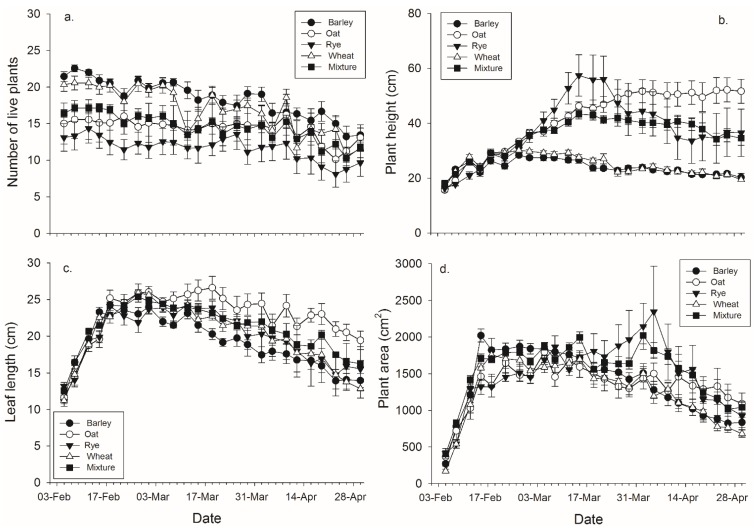
Mean (± SEM) (**a**) number of plants surviving within a pot; (**b**) height of the tallest plant per pot; (**c**) length of the leaves per pot, and (**d**) plant area in pots with each plant species and pots with mixtures.

There was not a significant main effect of banker plant treatment (monoculture *vs.* mixture) on the number of plants surviving within a pot. There was a significant effect of day since plants died over time, but not of the banker plant treatment by day interaction (Figure 1, Table 2). There was a significant effect of banker plant treatment, day, and their interaction on banker plant height wherein mixture pots had taller plants in the last half of the experiment. (Figure 1, Table 2). There was not a significant main effect of banker plant treatment on average leaf length. There was a significant effect of day since plants grew over time, but not of the banker plant treatment by day interaction on average leaf length (Figure 2, Table 1). There was significant main effect of banker plant treatment and day on banker plant area wherein mixtures had a greater area but all plants increased in area over time. The interaction was not significant (Figure 1, Table 2).

**Table 2:** Results of repeated measures ANOVAs testing the effect of diversity (monocultures *vs.* mixtures) and the effect of species or mixtures on banker plant growth characteristics.

Response	Treatment	Day	Treatment * Day
Monoculture *vs.* Mixtures			
Survival	*F*_1,70_ = 0.90, *p =* 0.346	*F*_24,1680_ = 30.35, *p <* 0.001	*F*_24,1680_ = 0.65, *p =* 0.902
Height	*F*_1,70_ = 5.12, *p =* 0.027	*F*_24,1662_ = 52.17, *p <* 0.001	*F*_24,1662_ = 1.64, *p =* 0.026
Leaf length	F_1,70_ = 1.45, *p =* 0.233	*F*_24,1662_ = 122.35, *p <* 0.001	*F*_24,1662_ = 0.99, *p =* 0.474
Area	*F*_1,70_ = 8.90, *p =* 0.004	*F*_24,1662_ = 54.18, *p <* 0.001	*F*_24,1662_ = 0.80, *p =* 0.736
Species and Mixtures			
Survival	*F*_4,67_ = 6.10, *p <* 0.001	*F*_24,1608_ = 23.26, *p <* 0.001	*F*_96,1608_ = 1.41, *p <* 0.001
Height	*F*_4,67_ = 17.20, *p <* 0.001	*F*_24,1590_ = 32.42, *p <* 0.001	*F*_96,1590_ = 6.60, *p <* 0.001
Leaf length	*F*_4,67_ = 11.50, *p <* 0.001	*F*_24,1590_ = 88.59, *p <* 0.001	*F*_96,1590_ = 3.82, *p <* 0.001
Area	*F*_4,67_ = 5.93, *p <* 0.001	*F*_24,1590_ = 103.91, *p <* 0.001	*F*_96,1590_ = 2.59, *p <* 0.001

There were significant effects of banker plant species, day, and their interaction on each of the four plant growth characters (Figure 2, Table 3). Barley had significantly more live plants than rye and the mixtures, while wheat had significantly more live plants than rye (Figure 2, Table 3). There were significant effects of species, day, and their interaction on the height of the tallest plant (Figure 2, Table 2). Oat, rye, and the mixtures were significantly taller than wheat and barley by the end of the experiment (Figure 2, Table 3). There were significant main effects of average leaf length, day, and their interaction (Figure 2, Table 2). Oat, rye, and the mixtures were significantly longer than barley, and oat was significantly longer than wheat and the mixtures, by the end of the experiment (Figure 2, Table 3). There were significant effects banker plant species, day, and their interaction on log transformed banker plant area (Figure 2, Table 2). Rye was significantly greater than wheat, while the mixtures were significantly greater than both oat and wheat by the end of the experiment (Figure 2, Table 3).

**Table 3:** Mean (± SEM) plants surviving, plant height, leaf length, and plant area of plant species and mixtures. Means followed by different letters within a column are significantly different using Tukey’s HSD after a significant main effect of plant species in repeated measures ANOVA (Table 2).

Plant species or mixture	Number of Plants	Height (cm)	Leaf Length (cm)	Plant Area (cm^2^)
Barley	18.5 ± 1.1 ^a^	23.8 ± 2.0 ^b^	19.0 ± 0.4 ^c^	1374.1 ± 91.3 ^ab^
Oat	14.3 ± 1.1 ^b^	41.3 ± 2.0 ^a^	22.6 ± 0.4 ^a^	1339.0 ± 147.2 ^b^
Rye	11.7 ± 1.1 ^c^	40.1 ± 2.0 ^a^	21.4 ± 0.4 ^ab^	1485.9 ± 210.2 ^a^
Wheat	16.9 ± 1.1 ^ab^	24.8 ± 2.0 ^b^	20.1 ± 0.4 ^b^	1259.9 ± 88.4 ^b^
Mixture	14.5 ± 0.5 ^b^	35.3 ± 1.0 ^a^	21.0 ± 0.2 ^b^	1531.5 ± 35.1 ^a^

### 3.2. Interspecific Grain Effects on Aphid and Parasitoid Population Growth

**Figure 3:**
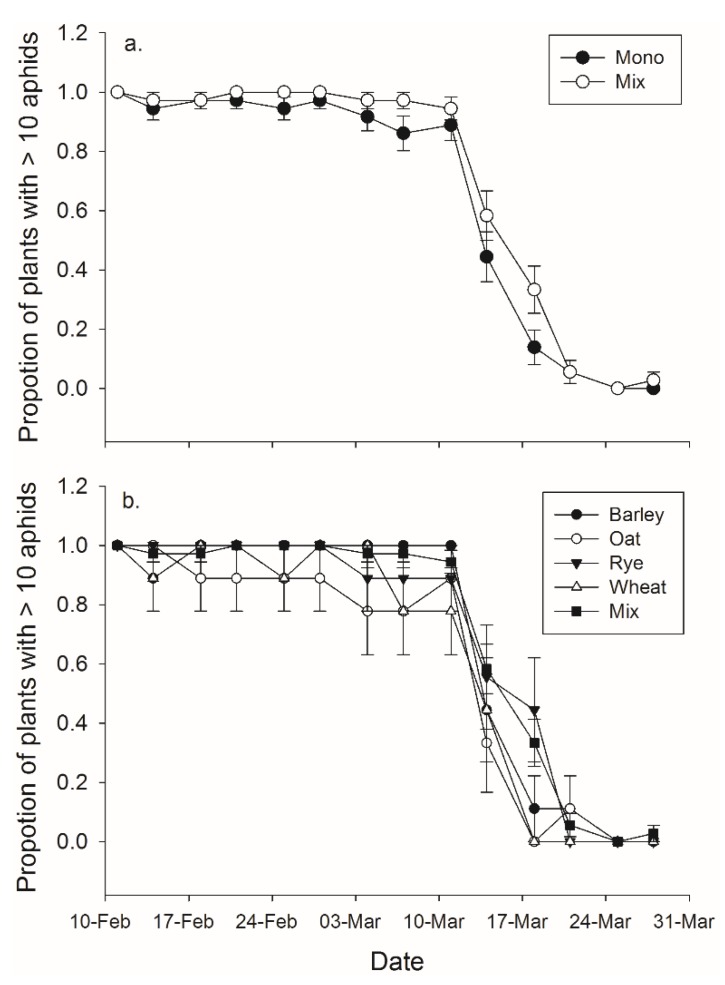
Mean (± SEM) proportion of pots which contained an efficient number of aphids—considered to be greater than 10—to function as a banker plant for (**a**) monoculture and mixture treatments and in (**b**) mixture and each of the four monoculture species.

There was a significant effect of both banker plant treatment and day on the effective duration of banker plants (*F*_1,70_ = 6.80, *p =* 0.011; *F*_13,910_ = 216.25, *p* *<* 0.001, respectively), but the interaction between day and treatment was not significant (*F*_13,910_ = 1.09, *p* *=* 0.365) (Figure 3). Banker plant species and day had significant effects on the effective duration of banker plants (*F*_4,67_ = 3.65, *p* *=* 0.010; *F*_13,871_ = 160.21, *p* *<* 0.001, respectively), but the interaction between day and treatment was not significant (*F*_52,871_ = 1.01, *p =* 0.452) (Figure 3).

**Figure 4:**
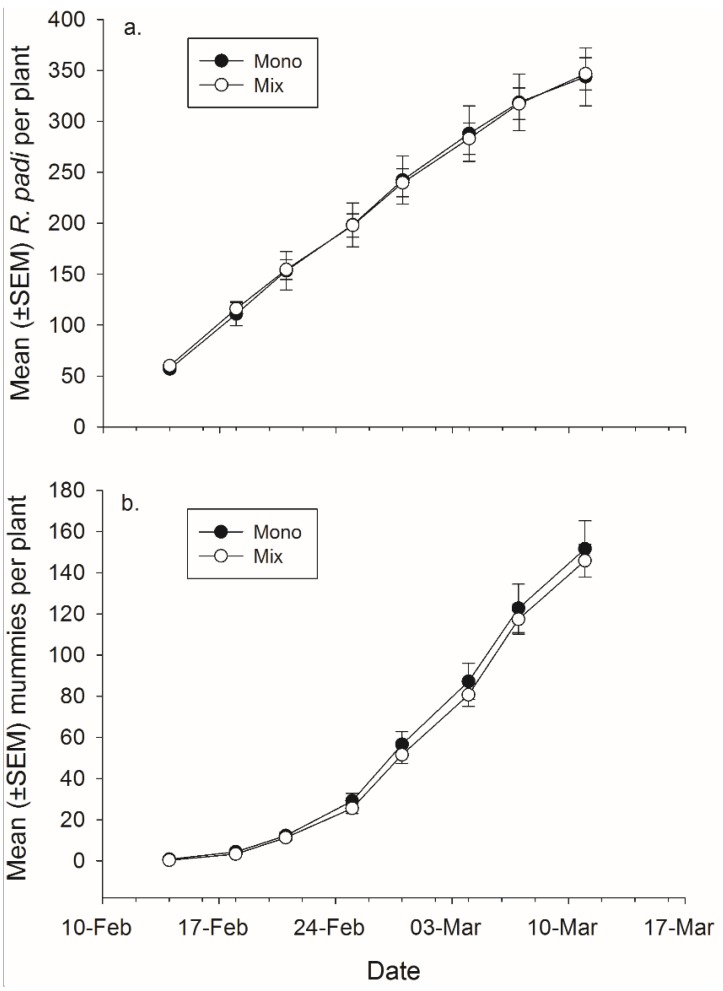
Mean (± SEM) total number of (**a**) *R. padi* produced and (**b**) mummies produced in monoculture and mixture grain pots on each data collection day.

There was not a significant effect of banker plant treatment on *R. padi* or mummy production (Figure 4, Table 4). There was a significant effect of day in both cases, as cumulative *R. padi* and cumulative mummy productions increased over time. There was not a significant interaction between plant mixture and day on either *R. padi* nor mummy production (Figure 4, Table 4).

**Table 4:** Results of repeated measures ANOVAs testing the effect of diversity (monocultures *vs.* mixtures) and the effect of species and mixtures on aphid and mummy abundance per banker plant.

Response	Treatment	Day	Treatment * Day
Monoculture *vs.* Mixtures			
Aphids	*F*_4,67_ = 4.70, *p =* 0.002	*F*_7,490_ = 1356.46, *p <* 0.001	*F*_7,490_ = 0.16, *p =* 0.992
Mummies	*F*_1,70_ = 0.17, *p =* 0.684	*F*_7,490_ = 1800.06, *p <* 0.001	*F*_7,490_ = 0.90, *p =* 0.503
Species and Mixtures			
Aphids	*F*_4,67_ = 6.10, *p <* 0.001	*F*_7,469_ = 1041.74, *p <* 0.001	*F*_28,469_ = 1.52, *p =* 0.045
Mummies	*F*_4,67_ = 5.12, *p =* 0.001	*F*_7,469_ = 1296.53, *p <* 0.001	*F*_28,469_ = 1.07, *p =* 0.372

**Figure 5:**
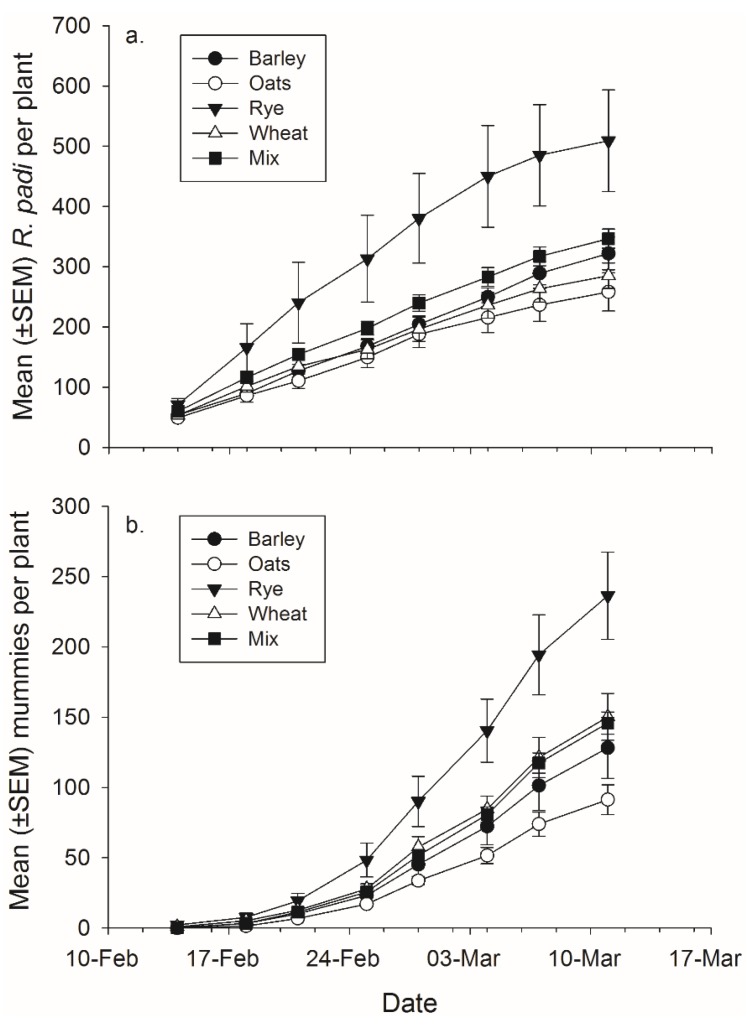
Mean (±SEM) total number of (**a**) *R. padi* produced and (**b**) mummies produced on mixtures and each plant species in monoculture.

Plant species significantly affected *R. padi* production (Figure 5, Table 4). Rye banker plants produced significantly more *R. padi* than any of the other species but not more than the mixtures, the difference between rye and barley is only significant at *p <* 0.06 (rye-barley: *t*_67_ *=* 2.75, *p =* 0.058; rye-oat: *t*_67_ *=* 3.83, *p =* 0.003; rye-wheat: *t*_67_ *=* 2.98, *p =* 0.032) (Figure 5). There was also a significant effect of plant species on mummy abundance. Rye had significantly more total mummies than barley and the mixtures but not wheat (rye-barley: *t*_67_ = 3.07, *p =* 0.025; rye-oat: *t*_67_ = 4.24, *p <* 0.001; rye-mixture: *t*_67_ = 3.10, *p =* 0.023) (Figure 5). There was a significant effect of day for both *R. padi* and mummy abundance as *R. padi* and mummy abundance increased over time (Figure 5, Table 4). There was no significant effect of the banker plant by day interaction for either *R. padi* or mummy production (Figure 5, Table 4).

### 3.3. Mixture Banker Plant Effects on Aphidius colemani Host Preference

**Figure 6:**
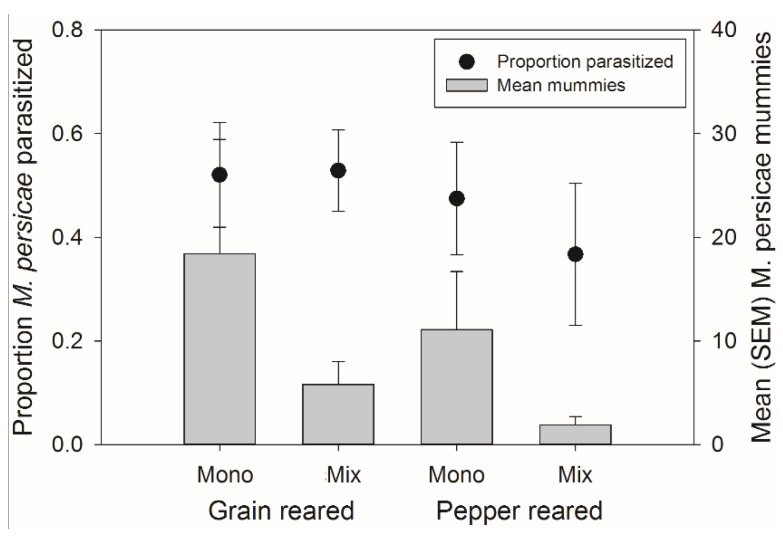
Mean (±SEM) percent of parasitism occurring on the crop (left axis, circles) and mean (±SEM) number of mummies on the crop (right axis, bars) from both natal habitats (Grain or Pepper reared) and both treatments (Monoculture or Mixture). There were no significant differences in the number of mummies or percent of mummies occurring on the crop plant.

There was no significant effect of rearing habitat, banker plant treatment, or their interaction on the percent of parasitism of *M. persicae* on the crop (*F*_1,54_ = 2.95, *p =* 0.092; *F*_1,54_ = 1.59, *p =* 0.213, and *F*_1,54_ = 0.00, *p =* 0.953, respectively) (Figure 6). There was also, no significant effect of rearing habitat, banker plant treatment, or their interaction on the number of mummies on the crop (*F*_1,54_ = 2.46, *p =* 0.123, *F*_1,54_ = 2.08, *p =* 0.155, and *F*_1,54_ = 0.00, *p =* 0.952, respectively) (Figure 6).

### 3.4. Effects of Banker Plant Mixtures on Aphidius colemani Pest Suppression and Fitness

**Figure 7:**
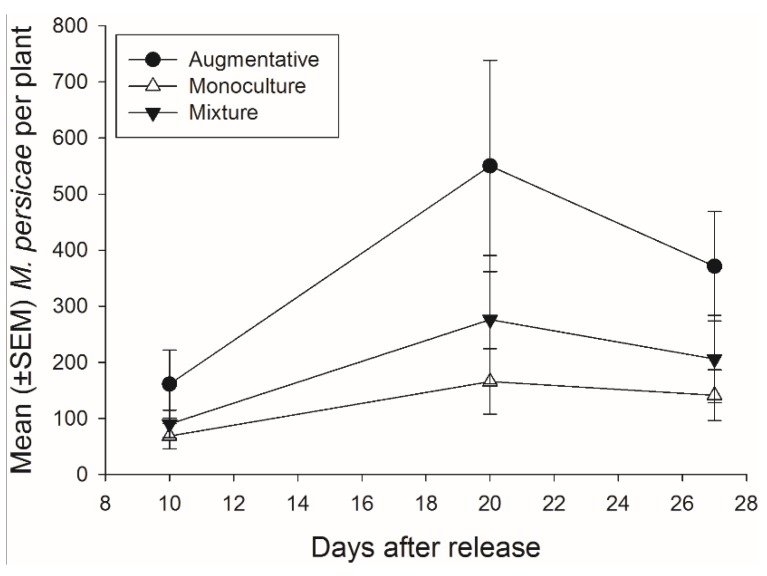
Mean (±SEM) number of pest aphids (*M. persicae*) on crop plants for each of the three treatments monoculture, mixture, and augmentative per collection day.

There was not a significant effect of banker plant treatment on *M. persicae* abundance during the 27 days of the experiment (*F*_2,92_ = 2.48, *p =* 0.089) (Figure 7). There was a significant effect of day, as *M. persicae* abundance increased and decreased over time, but there was not a significant interaction of banker plant treatment and day (*F*_2,92_ = 14.40, *p <* 0.001; *F*_4,92_ = 1.06, *p =* 0.381, respectively).

The percentage *M. persicae* in a cage was positively correlated with the percentage of mummies on the crop in a cage suggesting *A. colemani* prefer to oviposit on plants with denser aphid populations. This correlation was stronger in the monoculture banker plant treatment than mixture banker plant treatment for each day (day 10: monoculture *N* = 11, *p =* 0.020, *r* = 0.684, mixture *N* = 11, *p =* 0.153, *r* = 0.462; day 20: monoculture *N* = 11, *p =* 0.009, *r* = 0.740, mixture *N* = 11, *p =* 0.044, *r* = 0.614; day 27: monoculture *N* = 11, *p =* 0.005, *r* = 0.774; mixture, *N* = 11, *p =* 0.013, *r* = 0.716). There was a stronger correlation between percentage of *M. persicae* in a cage and the percentage mummies in a cage on the crop, for the monoculture treatment compared to the mixture treatment indicating the mixture banker plants may be altering this result.

For the per cage totals (both plants in each cage) we found a significant effect of treatment on frequency of females. The mixture treatment had the highest female frequency 0.65, followed by monoculture 0.56, and then the control 0.24 (overall χ^2^ = 44.34, *p <* 0.001, monoculture *vs.* control χ^2^ = 27.85, *p <* 0.001, mixture *vs.* control χ^2^ = 43.18, *p <* 0.001, and monoculture *vs.* mixture χ^2^ = 4.04, *p =* 0.044).

**Figure 8:**
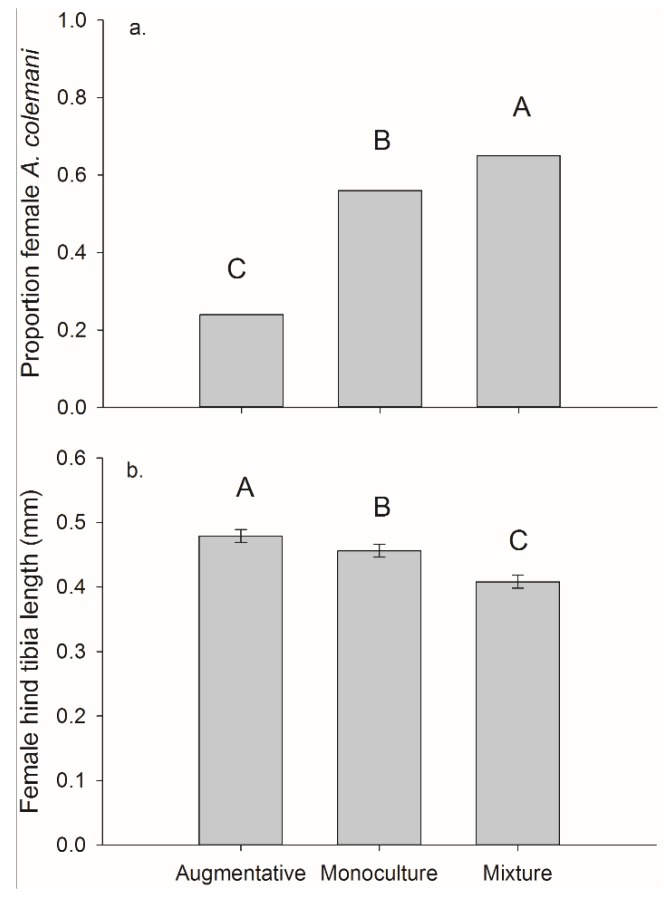
Mean (±SEM) (**a**) proportion of *A. colemani* that were female and (**b**) female hind tibia length of wasps reared from *M. persicae* on pepper plants in cages with augmentative release of *A. colemani*, monoculture banker plants, or mixture banker plants.

There was a significant main effect of banker plant treatment on female parasitoid size and on parasitoids collected on crop plants (*F*_2,181_ = 30.70, *p <* 0.001) (Figure 8). Female parasitoids collected from crop plants were significantly larger in the augmentative cages, hind tibia length 0.479 ± 0.007 mm than the other two treatments. The female parasitoids collected off crop plants in the monoculture cages, 0.456 ± 0.008 mm were significantly larger than those collected in mixture cages, 0.408 ± 0.007 mm (mixture-augmentative: *t*_181_ *=* −4.83, *p <* 0.001, mixture-monoculture: *t*_181_ *=* −7.70, *p <* 0.001, and monoculture-augmentative: *t*_181_ *=* −2.37, *p =* 0.019). There was no significant main effect of banker plant treatment on the size of female parasitoids collected off monoculture 0.462 ± 0.001 mm and mixture 0.468 ± 0.007 mm banker plants (*F*_1,116_ = 0.28, *p =* 0.595) (Figure 8).

## 4. Discussion

Diverse plant mixtures should increase plant growth [18,19,22]. Our study supports this since grain mixtures grew taller and had larger area than monocultures. Associational resistance suggests *R. padi* abundance should be lower on mixture banker pants compared to monocultures but this effect was not observed in our study [3]. There were other differences between the monoculture an mixture treatments, such that mixtures increased female frequency, but decreased female size of parasitoids emerging from crop plants, and decreased proportion of oviposition on crop pests. These differences did not affect the functionality of banker plants. We observed similar effective durations, number of aphids produced, number of mummies produced, *A. colemani* preference, and pest supression by *A. colemani* for both monoculture and mixture treatments. Interspecifc mixture banker plants are neither a benefit nor a detriment to the aphid banker plant system.

Growers prefer banker plants to be easy to cultivate, long-lasting, and to not compete with their crop plants for light, space, or nutrients [32]. Several different banker plant species have been used in aphid banker plant systems [4]. Jandricic *et al.* [14] found that the four plant species we tested each had different effects on aphid and parasitoid life history characters such as sex-ratio but were unable to identify a best banker plant species. Our open bench study suggests banker plants remain effective sources of parasitoids for the same duration regardless of species. Barley banker plants took up less space since they had shorter leaves and height than oat and rye and had a smaller area than rye. Barley pots also contained more plants than oat and rye pots which provide more basal stems and concealed locations that *R. padi* often prefer [33]. However, aphids will feed on all plant parts [34]. As such, rye banker plants, provided the most plant material and also produced the most mummies. Jandricic *et al.* [14] found rye to have less aphids and mummies than barley and wheat. Although we cannot fully explain these differences, we did use one different rye variety “Matt Time” and different methods than the previous study. Our study and Jandricic *et al.* [14], found that oat produced the smallest aphid and mummy populations. The total number of parasitoids released is a major factor in determining the success of augmentative releases [35,36]. As such, optimal banker plant species should produce as many parasitoids as possible over their effective duration, and our studies suggest that rye banker plants may be preferable to the other species we tested.

Regardless of species and whether grains are grown as monocultures or mixtures, all banker plants remained effective sources of parasitoids—which according to our definition meant the plants had more than 10 aphids—for four weeks. After four weeks the percentage of plants with more than 10 aphids dropped rapidly, such that after 4.5 weeks only around 50% of all banker plants were considered effective. By 5.5 weeks no banker plants more than 10 aphids because most of the aphids in each pot had been parasitized. Our experiment did not have crop plants present. Therefore, parasitism on the banker plants was likely greater and effective duration less than if *A. colemani* were also ovipositing in pest aphids. To our knowledge this is the first time effective banker plant duration has been evaluated. Our results suggest growers should inspect banker plants at least every four weeks to estimate aphid abundance.

For each banker plant species and mixtures in the open bench experiment, there was a positive correlation between *R. padi* and mummy production, which was expected as *A. colemani* oviposit more where host density is high [17,23]. We also observed a density dependent response in our pest suppression study, where the percentage of mummies on the crop increased as the percentage of *M. persicae* in a cage increased. Correlations in banker plant mixtures were not as strong as the monoculture treatment, which suggests something was drawing parasitoids in the mixture treatment away from the *M. persicae*. One potential explanation for this could be more or different volatiles given off by the mixture banker plants [25]. However, in our preference experiment each plant had 30 aphids. In this case *A. colemani* showed no preference for any of the four treatment combinations. This result is surprising since *A. colemani* often exhibits a preference for their natal host [37,38,39,40] and since *A. colemani* often prefer *M. persicae* to *R. padi*, regardless of natal host [6,37,41].

In our pest suppression study, monoculture and mixture banker plant cages both produced parasitoids with positive and negative life history characters. Although mixture banker plants produced a higher female frequency, females from pepper plants from monoculture cages were larger. Larger parasitoids generally have increased fitness and egg loads [8,14,42,43]. Thus, lower female frequency but larger female size in monoculture cages could result in a similar oviposition compared to the higher female frequency and smaller female size in mixture cages help explain why we observed similar pest suppression between the monoculture and mixture treatments.

In our pest suppression study, there were no differences between both banker plant treatments and the augmentative release. We believe our augmentative cages were able to perform similarly to the banker plant cages due to cage effects. One way banker plants are expected to improve biological control is by replacing parasitoids that disperse from the crop area. Since aphid abundance on the crop plants remained high and *A. colemani* were unable to disperse from the augmentative cages, pest suppression remained high without the aid of a banker plant. We note that we added *A. colemani* adults to some cages and mummies to others. An equal number of cages received adults or mummies in each treatment and there was no statistically significant effect of release type on the response variables we measured. However, it is possible this methodology caused unequal sex ratios among cages or had other effects on the outcome.

In open greenhouse experiments, banker plants have been more effective than augmentative releases in some cases [6,44,45]. In addition, banker plants often produce higher quality aphids than what is purchased in terms of female frequency and size [6]. We found that interspecific mixture banker plants are neither a benefit nor a detriment to the aphid banker plant system. Similar to other studies we found that rye and barley produce the more aphids and mummies than other species and may be the best choice of banker plants we have tested.

## 5. Conclusions

Our goal was to determine if banker plants made with species mixtures could improve biological control of *M. persicae*. Overall we found no consistent benefit of species mixtures compared to the best performing species. In this study rye produced the most plant material and the most mummies and, consistent with other studies, oats produced the least. Taken together our research suggests that growers can produce banker plants with mixtures or monocultures of any species except oats that are available in their region and that grow well under their particular greenhouse conditions. We suggest monitoring banker plants after 4–5 weeks to assess aphid and mummy abundance to maintain effective banker plants.
